# Gynecologic Cancer, Cancer Stem Cells, and Possible Targeted Therapies

**DOI:** 10.3389/fphar.2022.823572

**Published:** 2022-02-16

**Authors:** Vahideh Keyvani, Espanta Riahi, Meysam Yousefi, Seyed-Alireza Esmaeili, Rana Shafabakhsh, Amin Moradi Hasan-Abad, Maryam Mahjoubin-Tehran, Michael R. Hamblin, Samaneh Mollazadeh, Hamed Mirzaei

**Affiliations:** ^1^ Department of Biology, Faculty of Science, Shahid Chamran University of Ahvaz, Ahvaz, Iran; ^2^ Medical Genetics Research Center, Mashhad University of Medical Sciences, Mashhad, Iran; ^3^ Blood Borne Infections Research Center, Academic Center for Education, Culture and Research (ACECR), Mashhad, Iran; Department of Biology, Mashhad Branch, Islamic Azad University, Mashhad, Iran; ^4^ Department of Medical Genetics, School of Medicine, Ahvaz Jundishapur University of Medical Sciences, Ahvaz, Iran; ^5^ Immunology Research Center, Mashhad University of Medical Sciences, Mashhad, Iran; ^6^ Department of Immunology, Faculty of Medicine, Mashhad University of Medical Sciences, Mashhad, Iran; ^7^ Research Center for Biochemistry and Nutrition in Metabolic Diseases, Institute for Basic Sciences, Kashan University of Medical Sciences, Kashan, Iran; ^8^ Autoimmune Diseases Research Center, Kashan University of Medical Sciences, Kashan, Iran; ^9^ Department of Medical Biotechnology and Nanotechnology, Faculty of Medicine, Mashhad University of Medical Sciences, Mashhad, Iran; ^10^ Laser Research Centre, Faculty of Health Science, University of Johannesburg, Doornfontein, South Africa; ^11^ Natural Products and Medicinal Plants Research Center, North Khorasan University of Medical Sciences, Bojnurd, Iran; ^12^ Student Research Committee, Kashan University of Medical Sciences, Kashan, Iran

**Keywords:** gynecologic cancer, cervical cancer, cancer stem cells, ovarian cancer, endometrial cancer, molecular mechanism, carcinogenesis, chemoresistance

## Abstract

Gynecologic cancer is one of the main causes of death in women. In this type of cancer, several molecules (oncogenes or tumor suppressor genes) contribute to the tumorigenic process, invasion, metastasis, and resistance to treatment. Based on recent evidence, the detection of molecular changes in these genes could have clinical importance for the early detection and evaluation of tumor grade, as well as the selection of targeted treatment. Researchers have recently focused on cancer stem cells (CSCs) in the treatment of gynecologic cancer because of their ability to induce progression and recurrence of malignancy. This has highlighted the importance of a better understanding of the molecular basis of CSCs. The purpose of this review is to focus on the molecular mechanism of gynecologic cancer and the role of CSCs to discover more specific therapeutic approaches to gynecologic cancer treatment.

## Introduction

Cancer is a group of diseases associated with the abnormal growth of malignant cells and their expansion to other areas of the human body (Grever et al., 1992). Benign types of tumors are different from malignant tumor types and do not show the same metastatic activity. Cancer is caused by genetic and epigenetic modifications that enable uncontrolled cell growth, migration, and disturbances in cell death pathways. There are a number of molecular changes that drive the transformation of normal cells into malignant cells, but the spectrum and diversity of these changes vary widely among different cancer types (Spandidos et al., 2000).

Gynecologic malignancies, including ovarian, cervical, and endometrial cancer, seriously affect the health of women worldwide, contributing considerably to the global cancer burden. Epithelial ovarian cancer comprises ∼90% of malignant ovarian neoplasms, which is a leading cause of death in women. The 5-year overall survival (OS) rate of OC is ∼47% for all stages, and >70% of patients are diagnosed at an advanced stage with an even lower 5-year OS rate (Wang et al., 2020).

Currently, some potential therapeutic targets include tumor-intrinsic signaling pathways, angiogenesis, homologous recombination deficiency (HDR), hormone receptors, and immunologic factors. The corresponding targeted therapeutic agents include signaling pathway inhibitors, antiangiogenic agents, poly (ADP-ribose) polymerase (PARP) inhibitors, selective estrogen receptor downregulators, and immune checkpoint inhibitors (Wang et al., 2020).

In 1877, Virchow’s student Cohnheim discovered a new cell population in tumors and pointed out that it possessed an embryonic character (Spandidos et al., 2000). Today, those cells are called cancer stem cells (CSCs) or tumor-initiating cells (TICs) and are seen as drivers of tumor establishment and growth, often correlated to aggressive, heterogeneous, and therapy-resistant tumors. CSCs are a subpopulation of tumor cells that can drive tumor initiation and can cause recurrence after treatment. At the time point of tumor initiation, CSCs can originate from either differentiated cells or adult tissue-resident stem cells. Due to their importance, several biomarkers to characterize CSCs have been identified and correlated with diagnosis, prognosis, and response to therapy in patients. However, CSCs have been shown to display a high degree of plasticity, which changes their phenotype and functional properties. Such changes are induced by chemotherapy and radiotherapy, as well as the presence of senescent tumor cells, which cause alterations in the tumor microenvironment (Walcher et al., 2020).

Stem cell studies have provided scientists with useful information about understanding the contribution of the CSCs to cancer progression, resulting in the discovery of novel methods and therapeutic targets of CSCs (Meacham and Morrison, 2013). CSCs possess the capacity for self-renewal and can generate heterogeneous lineages of cancer cells within tumors. A substantial body of evidence supports a model in which CSCs play a major role in the initiation, progression, and clinical outcome of cancer. The initiation of cancer by CSCs is attributed to their stemness property, allowing them to accumulate underlying carcinogenic mutations including those related to inflammation and oxidative stress. CSCs can further promote cancer growth and progression by mutual interaction with the microenvironment, which allows them to favor their own survival, expansion, resistance to therapy, promotion of angiogenesis, and metastatic capability. Therefore, CSCs are potential therapeutic targets for the development of therapies that could control cancer and achieve improved clinical responses in patients (Ayob and Ramasamy, 2018).

The CSC model proposes that tumor initiation, growth, and progression are fueled and sustained by undifferentiated cancer cells endowed with self-renewal on the one hand and more differentiated cells on the other hand. Gynecologic malignancies, based on their biological behavior and clinical course, represent a typical example of CSC-driven cancer. For example, several markers such as CD133, ALDH1/2, LY6A, LGR5, EpCAM, CD133, CD44, CD34, CD24, CD117, MyD88, and CDH1 have been used for the isolation of CSCs from ovarian cancer cell lines. The most common signaling pathways activated by endometrial CSCs are Wnt/β catenin, Notch1, and Hedgehog. Targeting the Notch3 pathway (a transmembrane protein) is a novel method for possible eradication of ovarian CSCs. Gamma-secretase inhibitors (GSI) active against Notch receptors have been used in preclinical and clinical studies (Zhan et al., 2013; Moghbeli et al., 2014; Keyvani et al., 2019). The use of CSCs has been evaluated as vaccines, for example, in colorectal cancer (NCT02176746), hepatocellular cancer (NCT02089919), and pancreatic cancer (NCT02074046).

Traditional cancer therapies, including routine chemotherapy and radiotherapy, have major limitations, especially in eradication of CSCs, leading to frequent recurrence of the cancer mass. Therefore, targeting CSCs could be highly effective in preventing cancer recurrence (Dragu et al., 2015). In this regard, mastermind-like 1 (MAML1), a molecule with few side effects, may be used for targeting CD44^+^ CSCs *via* repressing the canonical NOTCH pathway in esophageal squamous cell carcinoma (ESCC) patients (Moghbeli et al., 2019).

CSCs are slow cycling and are capable of both self-renewal and differentiation. In the context of time-dependent tumor growth, CSCs actively participate in tumor mass expansion and morphogenesis. Many articles have shown that CSCs contain a spectrum of heterogeneous cell populations. These so-called “stem cell markers” can also change with time. Moreover, CSCs show a great variation in percentage composition across different tumor types. Thus, to claim that CSCs persist in a non-dividing state may not be accurate. Perhaps, CSCs can migrate to the bone marrow or other organs where they may remain in a dormant state for years.

The molecular mechanisms underlying gynecologic cancers, the specific molecular changes in these cancers, and the contribution of CSCs to the expansion and survival of these cancers are reviewed, in order to highlight the importance of targeting CSCs as a new therapeutic approach in the field of personalized medicine.

## Oncogenesis

At least 3–6 separate genetic modifications are needed to transform a normal cell into a cancerous cell (Croce, 2008). Most cancer cells are genetically unstable, and this instability results in the accumulation of numerous secondary molecular changes in the cell, which play important roles in the development of the malignant properties, like invasion, immortality, drug resistance, and metastasis (Hanahan and Weinberg, 2011). Genomic instability is associated with a higher frequency of mutations in the genome. These mutations can be very different and can include alterations in the sequence of nucleic acids, chromosomal rearrangements, or aneuploidy. Genomic instability has particular importance in multicellular organisms and can be the cause of neurological diseases like neurotoxic myotonic dystrophy or amyotrophic lateral sclerosis (Schmitt et al., 2012). This instability may be due to the higher frequency of external DNA damage followed by mutations caused by errors in the repair process or by incorrect translation. Other sources of genomic instability include mutational and/or epigenetic reductions in the expression of DNA repair genes. Endogenous DNA damage is common; indeed it occurs >60,000 times/day in the human cellular genome (Møller, 2005). Certain modifications in genes stimulating cell growth (oncogenes) may also lead to malignancy in normal cells (Doroshow and Kummar, 2014). Oncogenes are activated by different mechanisms. Upregulation of oncogenes can increase the expression of the proteins, while one-point mutations can lead to oncogene activation. In addition, oncogenes can be shifted from one chromosome to another and can be affected by the promoter region of the new site, resulting in the enhancement of oncogene expression (Negrini et al., 2010).

Tumor-related oncogenes can cause unscheduled cellular proliferation and also chromosomal and genomic instability. There are a number of treatments that can not only block the activity of oncogenes but also specifically target tumor cells. Nevertheless, some studies have shown the involvement of oncogenes in the rapid growth of certain tumor cells, but not all types of tumors. Furthermore, oncogenes and their target cells interact with each other through the induction of proliferation and developmental reprogramming of the epigenome, which contributes essentially to the expansion of tumors. Evidence suggests a possible initiation of tumorigenesis through stem cell reprogramming, which could be a new role of oncogenes in the tumorigenesis process (Vicente-Dueñas et al., 2013).

Another issue during tumorigenesis is the upregulation of cell rapid growth modulated through cell cycle-associated molecules like RB, TP53, cyclins, CDKs, and E2F (Harris and Levine, 2005). Cell cycle dysregulation is one of the most important events that could occur during tumorigenesis, in which cells become highly resistant to senescence and cell death. In contrast, malignant cells undergo a cellular senescence process during normal cell cycle progression (Collado and Serrano, 2010). Cell death has different types, defined by various morphological criteria, including apoptotic, necrotic, autophagic, or mitosis-associated cell death (Su et al., 2015).

Further information on the molecular basis of gynecologic cancer and therapeutic options, based on CSCs, will be addressed in the next section.

## Invasion, Metastasis, and Angiogenesis

Angiogenesis is a process that is crucial in cancer progression. During the tumorigenesis process, the balance between proangiogenic factors and angiogenic inhibitors becomes disturbed. One of the most important regulators of angiogenesis is vascular endothelial growth factor A (VEGF-A), which is involved in the progression of the epithelial ovarian cancer. Accordingly, some drugs have been developed including bevacizumab (a monoclonal anti-VEGF antibody) to inhibit angiogenesis during cancer progression (Burger et al., 2011). In another therapeutic approach, neoadjuvant chemotherapy after interval debulking surgery has been used to manage gynecologic cancer, especially advanced ovarian malignancies (Chéreau et al., 2013). Some studies have reported that the expression and activity of matrix metalloproteinases (MMPs) are important in several human cancers. These proteins contribute to invasion, metastasis, and the advanced stage of the tumor (Hua et al., 2011). MMP-9 and MMP-2 were increased in invasive endometrial cancer, and the high amounts of MMP-9 and MMP-2 colocalized with ETV5/ERM and RUNX1/AML1 (factors associated with neoplastic progression) (Planagumà et al., 2011). Another event that may occur during cancer development involves changes in cell–cell adhesion molecules, resulting in increased susceptibility of cells to exfoliation. One of the main molecules contributing to the adherence of adjacent cells is E-cadherin, a glycoprotein located at the cellular adherent junctions. In ovarian cancer, the expression level of E-cadherin in floating cancer cells in ascites and in metastatic deposits was lower than that in primary ovarian tumors (Sawada et al., 2008). The “seed-and-soil” hypothesis to explain the metastasis process was first attributed to Stephan Paget (1889), who proposed that circulating cancer cells (seeds) were only able to metastasize to organs where the microenvironment was particularly suitable for their growth and development (Zhang et al., 2010).

C-X-C chemokine receptor type 4 (CXCR4) (also known as CD184) is a diagnostic marker in several cancers, including breast cancer. The *CXCR4* gene encodes a receptor protein located in the cell membrane, through which signaling pathways are activated, followed by regulation of cell proliferation. Current advances in cancer biology have highlighted the crucial role of CXCR4 receptor and its respective ligand CXCL12, in the metastasis of different kinds of cancer (Mukherjee and Zhao, 2013). In different human cancers, the expression of the estrogen-responsive gene *RCAS1* has been correlated to clinical outcomes in ovarian cancer and to the survival rate in patients suffering from uterine and cervical adenocarcinoma, as well as esophageal, pancreatic, lung, gallbladder, and pancreatic cancers. Researchers have also shown a relationship between the expression of *RCAS1* and the invasion and metastasis of cervical, stomach, skin, breast, and thyroid cancers. *RCAS1* contributes importantly to the aggressive behavior of several kinds of cancer (Sonoda et al., 2005). Moreover, the transcription factor Snail potentially contributes to the epithelial–mesenchymal transition (EMT) process and to cancer growth, metastasis, and invasion. It has been shown that MMP expression in mammary epithelial cells can stimulate Snail expression and subsequently the EMT in cancer cells (Przybylo and Radisky, 2007). Moreover, *RCAS1* promotes angiogenesis and accelerates tumor growth in immune-deficient nude mice. Taken together, the data suggest that *RCAS1* affects the tumor–stroma interaction to enhance angiogenesis and is crucial for tumor growth *in vivo*.

The expression of VEGF can be affected by *RCAS1*, thus stimulating angiogenesis, endothelial cell motility, and vascular permeability (Sonoda et al., 2007). Moreover, the transforming growth factor-β (TGF-β) contributes to cancer progression, angiogenesis, escape from immunosurveillance, and myofibroblast recruitment. PI3K or phosphoinositide 3-kinase is a family of the enzymes that contributes to cellular functions like proliferation, motility, differentiation, intracellular trafficking, and cell survival. Previous studies have shown that the expression of PI3K is increased in ∼40% of ovarian cancers, while the expression and activity of its downstream effectors, AKT2 and AKT1, are higher in diverse cancers, such as ovarian tumors. In several kinds of tumor cells, AKT1 increases the stability of hypoxia-inducible factor, HIF-1α (Liby et al., 2012). During the angiogenesis process, researchers have observed the degradation of the basement membrane matrix by proteinases, among which the membrane-type matrix metalloproteinase (MT-MMP) is the most important example. As the angiogenesis process develops, the extracellular matrix (ECMs) proteins affect signaling cascades that contribute to proliferation, invasion, migration, and survival (Davis and Senger, 2005).

In Table 1, the changes in genes and proteins in different kinds of gynecologic cancers are summarized. In the following sections, the main molecular mechanisms involved in female genital malignancies are discussed to provide more information to choose the best therapeutic approaches.

**TABLE. 1:** Proteins and genes that have roles in gynecologic malignancies.

Gene/protein	Normal function	Function in cancer	Level	Cancer/s
VEGF-A	Growth factor	Angiogenesis and progression	High	Epithelial ovarian cancer
MMPs (MMP2/MMP9)	Degradation of the extracellular matrix	Increase invasion, stimulate Snail expression, and promote angiogenesis	High	Endometrial cancer
E-cadherin	Cell–cell adhesion molecule	Facilitate cell migration	Low	Ovarian tumors
			High	Floating cancer cells
CXCR4	Growth and division	Promote metastasis	High	Breast cancer
CXCL12	Ligand of CXCR4	Promote metastasis	High	Various cancers
RCAS1	Estrogen-responsive gene	Progression and invasion, promote angiogenesis, and stimulate VEGF expression	High	Various cancers
Snail	Transcription factor	Promote EMT process	High	Various cancers
TGF-β	Growth factor	Progression, angiogenesis, and immunosurveillance	High	Various cancers
PI3K/Akt1/Akt2	Enzymes involved in cellular functions	Cell survival	High	Ovarian cancer
ECM	Provide physical scaffolding, define tissue morphogenesis	Migration, invasion, proliferation, and survival	High	Various cancers

## Gynecologic Malignancies

### Cervical Cancer

As mentioned earlier, CC is the fourth most common cause of the cancer death in women throughout the world (Cancer IAfRo, 2003). In 2012, approximately 528,000 CC patients were reported, of whom 266,000 died, accounting for about 8% of all deaths from cancer. The human papillomavirus subtypes HPV18 and HPV16 are the most prevalent types of carcinogenic HPV and are responsible for 70% of CC cases, as well as 50% of cervical intraepithelial neoplasia grade 3 (CIN3) (Schiffman et al., 2007). Differences in the pathogenicity of HPV oncogenic variants (OTs) compared to non-oncogenic variants (NOTs) are caused by DNA sequence changes which have occurred over millions of years and are still evolving (Burk et al., 2009). The primary open reading frames (ORFs) encode seven proteins, called E1-7, which contribute to host cell transformation and viral replication. The E5, E6, and E7 oncoproteins are key viral factors that cause the initiation and expansion of CC and are largely responsible for HPV-related cancers by inducing genomic instability (Moody and Laimins, 2010). Over the past decades, it has been found that both HPV E7 and E6 can interact with numerous host proteins involved in apoptosis and malignant cell transformation. For example, HPV16/E6 regulates p53 protein activity by enhancing its degradation *via* a ubiquitin-dependent proteolytic pathway and subsequent proteasome-mediated degradation (Mammas et al., 2008).

Some studies have shown that tumors have a higher chromosomal imbalance in advanced stages. Common chromosomal imbalances include gain of 1q (36.7%), 8q (20.0%), and 3q (46.7%), as well as loss of 3p (33.3%), 11q (36.7%), 2q (20.0%), and 6q (23.3%). Moreover, the frequency of chromosomal imbalance in stage IIB or IB tumors was not remarkably different (Huang et al., 2007).

*WWOX* and *FHIT* are tumor inhibitor genes located in common chromosomal fragile sites FRA3B and FRA16D, respectively. FHIT is located on chromosome 3p, and its expression is clearly lower in CC. Analysis of the *FHIT* gene in CC showed a higher frequency of aberrant mRNA transcripts and alleles, whereas the fragile site of FRA3B is a candidate region for the integration of HPV16. These data suggest that the inactivation of the *FHIT* gene plays a critical role in the development and progression of CC (Giarnieri et al., 2010).

In addition, members of the RAS protein family are small GTPases that contribute to the signal transduction, and *K-RAS* or *H-RAS* mutations are a key event in CC (Spandidos et al., 2000). Current studies have reported a mutation in the *RAS* gene in CC, and although the overall RAS mutation frequency in gynecologic cancer is low, mutations in this gene occur more frequently at codon 12 (Spandidos et al., 2000; Harris et al., 2003). The tumor antigen 22-1-1 Ag has been found to occur in several cancers. One immunohistochemical study reported the expression of 22-1-1Ag in 87.5% of cervical and uterine cancers and in 58.8% of ovarian cancers (Sonoda et al., 1998). Table 2 provides a summary of some of the molecular changes that have been reported in CC.

**TABLE. 2:** Some factors involved in different type of cervical malignancies.

Cancer type	Percent	Factor/s	Event/s	Consequences
Cervical cancer	70%	HPV16 and HPV18 virus	Genomic integration	Induce genomic instability and promote P53 protein degradation
Cervical intraepithelial (neoplasia) grade 3	50%	HPV16 and HPV18 virus	Genomic integration	Genomic instability and promote P53 protein degradation
Cervical cancer	NA	FHIT gene	Deregulation/deactivation	Cancer development and progression
Cervical cancer	NA	Ras gene	Mutation	Cancer development and progression

### Endometrial Cancer

EC is one of the most common malignancies in women in developed countries (Wright et al., 2012). This cancer may be caused by microsatellite instability and mutations in *PIK3CA*, *PTEN*, *CTNNBI.*3, and *K-RAS* genes*.* One of the main factors that can increase the risk of EC is a genetic predisposition to obesity (Amant et al., 2005; Morice et al., 2016). Furthermore, Lynch syndrome is caused by a mutation in one of five different genes and has a higher risk of EC. Moreover, women with a genetic susceptibility to Lynch syndrome had a higher risk of EC than colon cancer. These patients showed a cumulative risk of 27–71% for endometrial cancer compared to only 3% risk in the general population (Meyer et al., 2009).

In addition, the risk of EC in women with mutations in *MSH2* or *MLH1* is between 27 and 60%, and for women with MSH6 mutations, it is similar. There are two clinicopathologic types of EC: estrogen-dependent type (i.e., type I, endometrioid carcinoma) and non-estrogen-dependent type (i.e., type II, non-endometrioid carcinoma). In the estrogen-dependent variant, microsatellite instability and mutations are observed in *PIK3CA*, *PTEN*, *CTNNB1* (β-catenin), and *K-RAS* genes, while in the non-estrogen-dependent variant, *TP53* gene mutations and chromosomal instability have been detected. There are studies that have suggested an essential role for non-coding RNAs in EC tumor progression (Matias-Guiu et al., 2001; Matias-Guiu and Prat, 2013).

In endometrial carcinogenesis, the different effects of estrone (E1), 17B-estradiol (E2), and estriol (E3) have been extensively documented. For example, E1 and E2 promote endometrial cell growth, whereas E3 selectively affects vaginal and uterine cervical cells without causing any endometrial proliferation (Niwa et al., 1993). In about 80% of endometrioid cancers, researchers have observed the loss of *PTEN* gene expression, while mutations in β-catenin and *K-RAS* genes have been repeatedly observed (Yeramian et al., 2013; Androutsopoulos et al., 2015). In sporadic endometrial cancers, various rates (9%–43%) of microsatellite instability (MSI) have been reported, while the phenotype of replication error repair (RER) has been observed in ∼45% of EC cases (MacDonald et al., 2000). About 20% of sporadic ECs show the MSI molecular phenotype. Inactivation of the tumor inhibitor gene *PTEN* is the most common genetic defect observed in EC, and in up to 83% of the tumors, it causes a histologically defined premalignant phase. In addition, researchers have identified a mutation in the *K-RAS* gene in 10–30% of estrogen-dependent types of EC. However, a much higher frequency of *K-RAS* gene mutations in MSI cancers has been described as the methylation-associated GC3AT transition (Hecht and Mutter, 2006). In EC, nearly 45%, 70%, and 80% of cases were, respectively, attributed to the inactivation of p16, overexpression of HER2, and reduced expression of E-cadherin (Halperin et al., 2001; Holcomb et al., 2002). In EC, the expression of the *RCAS1* gene was strongly associated with malignancy and poor differentiation (Sonoda et al., 2000). The expression of this gene has also been correlated to tumor progression or invasion in EC, CC, gastric, skin, and hepatocellular cancers. Thus, the *RCAS1* gene plays a key role in tumor invasion in humans, and its overexpression in EC carcinogenesis has been reported (Sonoda et al., 2003). In Table 3, we summarize some molecular factors related to different types of EC.

**TABLE. 3:** Some factors involved in endometrial malignancies.

Cancer type	Factor/s	Increased risk
Endometrial cancer	Lynch syndrome	27%–71%
Endometrial cancer	MLH1 and MSH2 gene mutations	27%–60%
Endometrial cancer	MSH6 mutation	27%–60%
Estrogen-dependent endometrial cancer	PTEN, PIK3CA, K-RAS, and CTNNB1gene mutations	Up to 80%
Non-estrogen-dependent endometrial cancer	TP53 gene mutation and chromosomal instability	NA
Sporadic endometrial cancer	MSI from 9% to 43%	Up to 45%
Sporadic endometrial cancer	RER (replication error repair) phenotype	Up to 45%
Endometrial cancer	Inactivation of the PTEN gene	Up to 80%
Estrogen-dependent endometrial cancer	Mutations in the K-ras gene	10–30%
Endometrial cancer	p16 inactivation	45%
Endometrial cancer	HER2 overexpression	70%
Endometrial cancer	Reduced E-cadherin expression	80%
Endometrial cancer	RCAS1 gene overexpression	NA

### Ovarian Cancer

Like many cancers, various genetic alternations have been reported to be involved in OC (Landen et al., 2008). Recurrence after remission is a challenging issue in most OC patients, resulting in treatment failure and resistance to chemotherapy. Reports have suggested that tumor heterogeneity often contributes to treatment failure (Gui et al., 2015). *BRAF* or *K-RAS* mutations have been shown to be common in borderline tumors, which are not seen in invasive serous carcinomas, and are only rarely found in other types of invasive tumors. These mutations affect different pathological pathways in these types of tumors (Mayr et al., 2006). In the OC overexpression of *HER-2/neu*, *AKT2*, and *MYC* genes, as well as frequent mutations in *TP53* have been observed (O'Neill et al., 2005; Nowee et al., 2007). OC, ovarian endometrioid carcinoma (EC), and clear cell ovarian carcinoma (CCC) have been associated with multiple mutations. Endometriosis shows mutations in the *ARID1A* tumor inhibitor gene as well as frequent mutations in *PIK3CA* and *PTEN* (Wiegand et al., 2014).

Mucinous epithelial ovarian cancer (mEOC) is a very rare type of OC. The fundamental clinical differences between mEOC and serous cancers are reflected by clinical differences, including an increased incidence of *K-RAS* mutation in mEOC, and especially in gastrointestinal tumors (Sonoda, 2016). About 10% of OC is caused by inherited mutations in cancer-susceptibility genes like *BRCA2* and *BRCA1*. Those women with inherited mutations in *BRCA2* and *BRCA1* show the highest risk of developing OC in their lifetime. There are also many environmental and epidemiological factors that have a significant effect on the incidence of both *BRCA* mutations including parity, ovulation, and hormone regulation (George and Shaw, 2014). *BRCA1* and *BRCA2* are located on chromosomes 17q and 13q (Powell and Kachnic, 2003). Instability in the chromosomal structure and inactivation of *BRCA1* and *BRCA2* lead to disturbances in mitosis and DNA repair (Venkitaraman, 2009). Moreover, the lifetime risk for OC is 10–20% and 20–40% for carriers of *BRCA2* and *BRCA1* mutations, respectively. Moreover, the average age of OC patients is about 40–50 years (Risch et al., 2001; Antoniou et al., 2003; McLaughlin et al., 2007). A group of active therapeutic agents for OC is *PARP* inhibitors, which cause DNA repair defects. Studies have shown that about 50% of high-grade serous cancers (HGSCs) have a deficiency in the DNA repair pathway. This may be due to abnormalities in somatic or germ line *BRCA*, posttranslational alterations of the *BRCA* gene, or disorders in other related molecules and factors (Milanesio et al., 2020).

In addition, many aberrant DNA pathways have been identified in histological subtypes of OC (Liu et al., 2014). PARP inhibitors have been proposed to increase progression-free survival in patients with platinum-sensitive, as well as relapsed and high-grade serous OC (Ledermann et al., 2012). It has been reported that the level of lysophosphatidic acid (LPA) in the plasma and ascites of OC patients is elevated at all stages. OC cell lines show some properties dependent on LPA signaling, such as cell adhesion/binding, generation of proangiogenic factors like interleukin-8 (IL-8) and VEGF, urokinase secretion, inhibition of apoptosis, and resistance to cisplatin-induced cell death (Fujita et al., 2003). Therefore, LPA has been proposed to be an important contributor to malignant behavior in OC and could be used as a new therapeutic target. In Table 4, some important genes are summarized, which affect the occurrence and progression of OC.

**TABLE. 4:** Genes and their alterations in ovarian cancer and progression.

Cancer type	Gene/s	Occurrence
Ovarian cancer	TP53, ARID1A, PTEN, and PIK3CA	Mutations
Ovarian cancer	HER-2/neu, AKT2, MYC	Overexpression
CCCs	ARID1A, PTEN, and PIK3CA	Mutations
Ovarian endometrioid carcinoma (EC)	ARID1A, PTEN, and PIK3CA	Mutations
Mucinous epithelial ovarian cancer	K-RAS	Mutations
Inherited ovarian cancer	BRCA1 and BRCA2	Mutations

The aggressive behavior of gynecologic tumors, their resistance to different cancer treatments, and the overall patient mortality rate could all be influenced by CSCs in the tumors. CSCs could provide not only a novel insight into gynecologic cancer biology but also lead to more precise targets for cancer treatment (Kenda Suster and Virant-Klun, 2019).

## Cancer Stem Cells in Gynecologic Cancer

About 20% of all visceral cancers in women are malignancies of the female genital tract. These cancers are the sixth main cause of mortality in women. Siegel et al. (2012) predicted that about 9% of malignancies occurring in developed countries were 3% in the ovary and 6% in the uterine body. In 2012, the death rate was 3 and 6% for uterine and ovarian cancers, respectively. About 80% of the gynecologic cancers are diagnosed in advanced stages, typically with visceral or peritoneal metastasis that is accompanied with higher death rates (Lengyel, 2010).

### Cervical Cancer Stem Cells

The intratumor genetic heterogeneity in CC has shown to be correlated with a poor response to radiotherapy and chemotherapy, as well as to pelvic recurrence and lymph node metastasis. As a result of the asymmetrical division of CSCs, CC tissue contains a variety of differentiated cancer cells (Kidd and Grigsby, 2008; Cooke et al., 2011). Cisplatin-based chemotherapy is a well-known treatment for CC. However, cervical CSCs (characterized by multiple markers) have been shown to be responsible for cisplatin resistance and radiotherapy resistance (López et al., 2012; Casagrande et al., 2013; Liu and Zheng, 2013). Researchers have investigated the complexity of the molecular mechanisms of cisplatin resistance (CPR), the most important of which is the decreased intracellular accumulation of the drug. Decreased drug uptake may also be due to the downregulation of copper transporter 1 (CTR1) or overexpression of drug efflux pumps, especially multidrug resistance protein (MRP1) and p-glycoprotein (P-gp, ABCB1). Moreover, cisplatin inactivation by thiol-containing proteins, such as GSH (a thiol-containing tripeptide Glu-Cys-Gly) can reduce the rate of cisplatin–DNA adduct formation (Shen et al., 2012). Tumor cells with acquired CPR have the potential to tolerate cisplatin-induced DNA lesions better than their parental cisplatin-sensitive counterparts. CPR can be caused by increased DNA excision repair activity. Nucleotide excision repair (NER) could be attributed to the upregulation of ERCC1, while mismatch repair (MMR) could be caused by the downregulation of PMS2 in human CC tissue or PMS2 overexpression as observed in HeLa cells (Galluzzi et al., 2012; Torii et al., 2014). Moreover, CRP development can be caused by the disruption of some proteins, including p53 and Bcl-2 families, or numerous other signaling pathways, such as nuclear factor-κB (NF-κB) and mitogen-activated protein kinase (MAPK) (Zhu et al., 2016). MicroRNAs also regulate several pathways contributing to the cellular response to cisplatin (Drayton, 2012). For instance, expression of miR-181 family members was increased in KB-CP20 and KBR-CP5 cells in comparison to the parental KB-3-1 cells (Pouliot et al., 2013). Furthermore, CSCs have been confirmed to have high resistance to a variety of chemotherapeutic drugs, which is due to very high expression levels of several drug resistance transporters, such as MDR1 and P-gp (Dean et al., 2005).

### Endometrial Cancer Stem Cell

Chemotherapy or hormonal therapy and combined chemo-hormonal regimens play an important part in the management of recurrent and advanced EC (Rauh-Hain and Del Carmen, 2010). Most patients with recurrence show inherent treatment resistance of some primary tumor cells, while early stages show better responses to surgery, hormonal therapy, and chemotherapy. In addition, Fas-mediated apoptosis is crucial for the normal endometrial cycling/remodeling. Available evidence suggested the contribution of dysregulation of Fas/FasL interactions to the initiation and progression of EC. Mutations, such as single-nucleotide polymorphisms (SNPs) in the *Fas* gene in its promoter area, also occur in EC (Chaudhry and Asselin, 2009). Sadarangani et al. (2007) have shown that TNF-related apoptosis-inducing ligand (TRAIL) can eliminate cancer cells without affecting normal cell apoptosis in reproductive malignancies. In addition, a study by Kato et al. showed cancer cell apoptosis could be caused by the estrogen metabolite 2-methoxyoestradiol alone, or combined with TRAIL without damage to normal cells. Therefore, TRAIL could be a practical agent for EC treatment (Kato et al., 2007). In another study, Chen et al. have shown that c-FLIP (FADD-like IL-1β-converting enzyme-inhibitory protein) was involved in carcinogenesis and invasion of EC and could also act as a prognostic marker. Increased FLIP expression contributes to chemotherapy resistance, while cisplatin remarkably reduces FLIP protein levels (Chen et al., 2005).

### Ovarian Cancer Stem Cells

**FIGURE 1:**
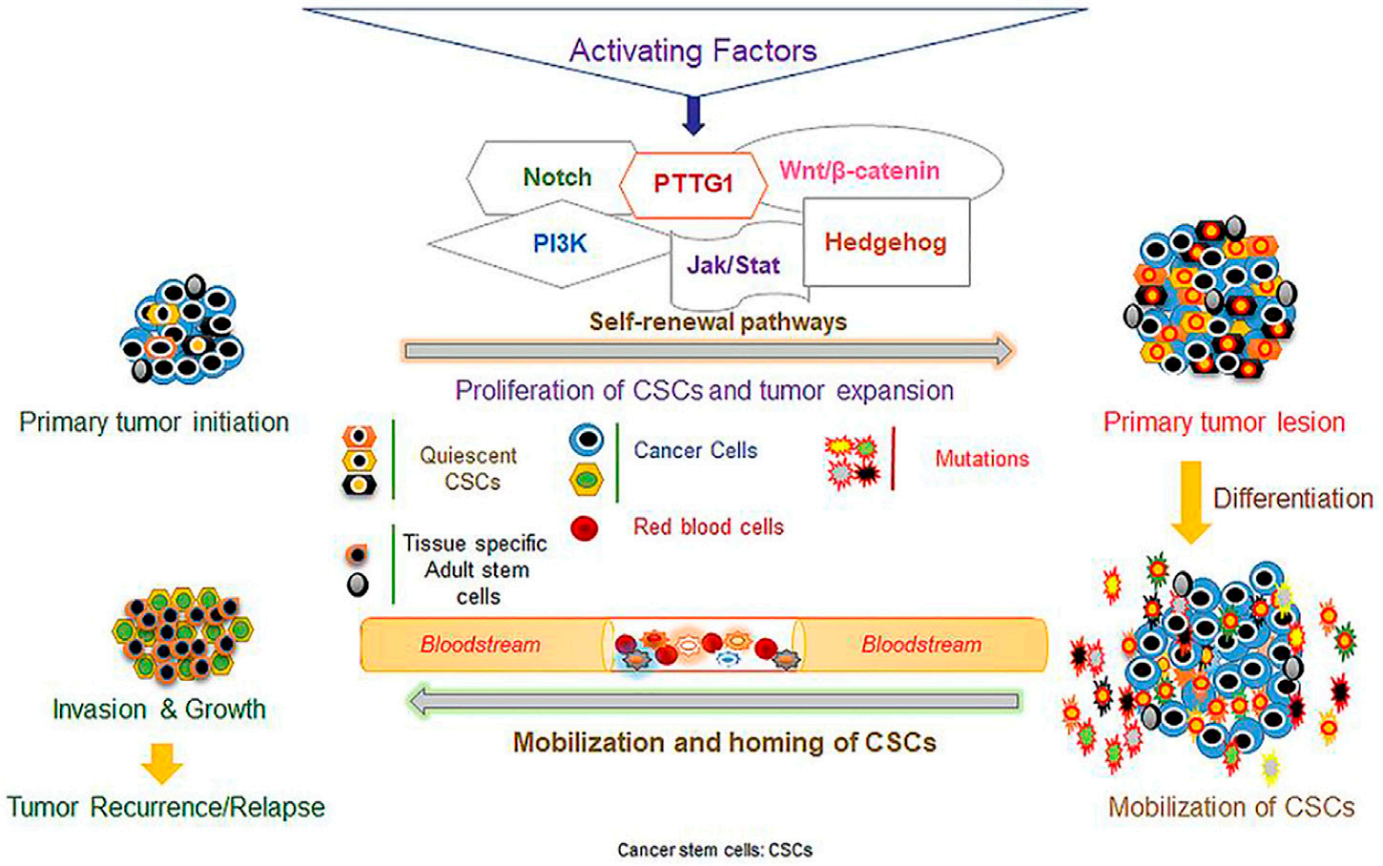
Different factors and signaling pathways mediating stem cell self-renewal, differentiation, and metastasis. (The figure was reprinted from Zuber et al.’s (2020) study.)

Researchers have shown that epithelial OC can metastasize to adjacent organs and seed the surfaces of organs in the peritoneal fluid (Lengyel, 2010). In fact, 90% of the tumors originating from the ovarian surface epithelium show the presence of the stem cells originating from this organ. During the initial stages of OC, it is possible to use the presence of EOC stem cells to predict the development of the disease (Zhan et al., 2013). Large numbers of EOC stem cells strongly predict the recurrence of the tumor. These observations have motivated the investigation of the molecular mechanisms that contribute to the escape of CSCs from chemotherapy (Ahmed et al., 2010; Hu et al., 2010). Researchers have confirmed the involvement of GSH-dependent enzymes and GSH in the development of chemoresistance in different tumors, such as brain tumors and OC (Kamazawa et al., 2002). Bmi-1 (polycomb complex protein) is an oncogene which allows cancer cells to escape apoptosis through several signaling pathways involved in cell growth. Thus, it is possible to use the increased expression of the Bmi-1 gene in cancer cells as a survival marker (Liu et al., 2009). Researchers have also shown that a loss of p53 function can result in multidrug resistance in various tumors including OC (Wallace-Brodeur and Lowe, 1999). Drug efflux from cancer cells is mediated by transmembrane multidrug efflux pumps that lower the intracellular content (Gillet and Gottesman, 2010). Therefore, the resistance mechanism of CSCs is important to understand the clinical progression in patients after apparently successful treatment (Arai et al., 2004). In Figure 1, the properties of CSCs regarding self-renewal, differentiation, and metastasis involving different factors and signaling pathway are shown.

## Gynecologic Cancer Stem Cell-Targeted Therapy

**FIGURE 2:**
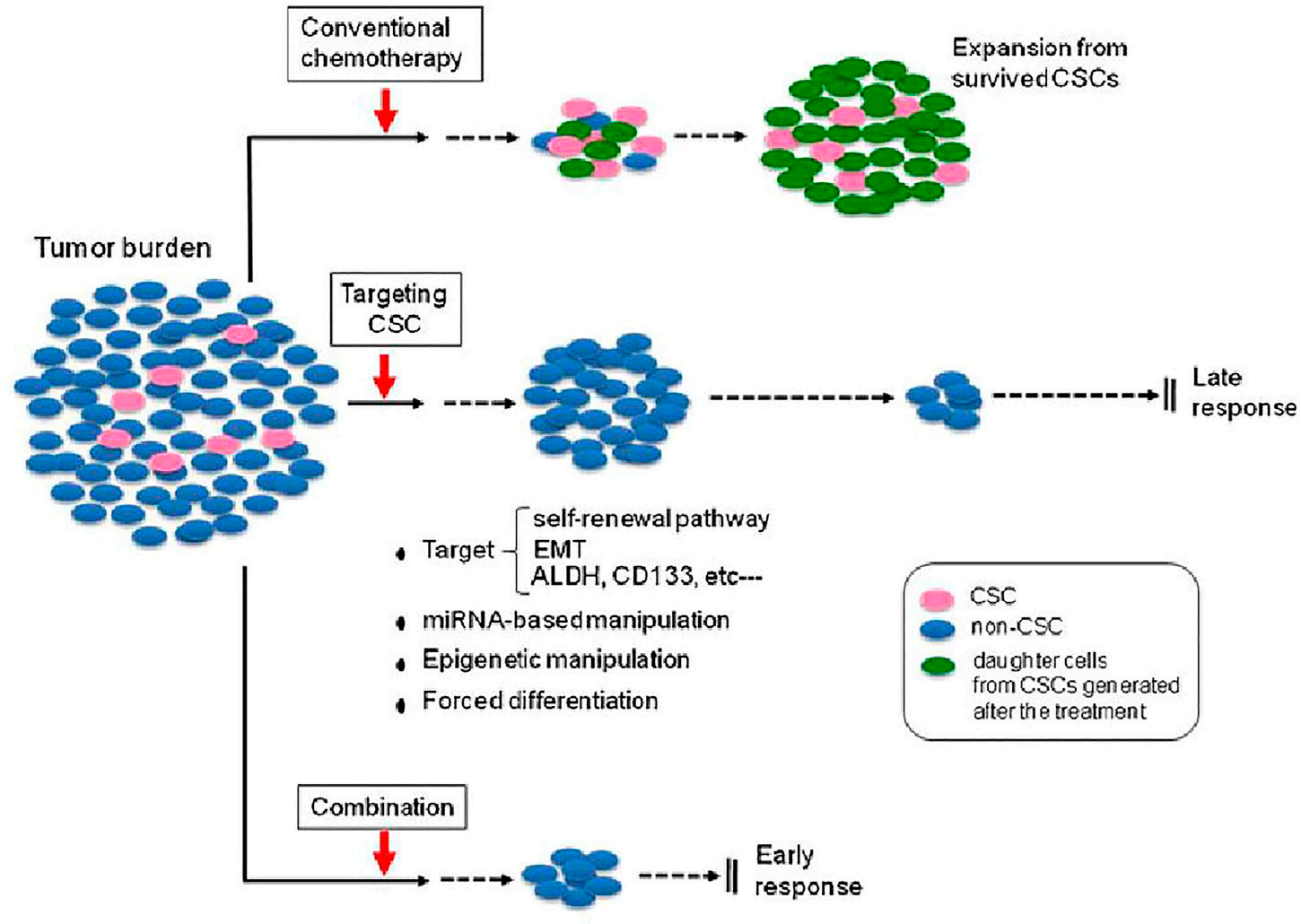
Effectiveness of targeted CSC therapy in combination with conventional chemotherapy. Conventional chemotherapy can decrease tumor size by acting on non-CSC tumor cells; however, tumor recurrence associated with CSCs may lead to treatment failure. Targeting the CSCs could reduce numbers of CSCs followed by better response of tumors to therapy. (The figure was reprinted from Kyo’s (2013) study.)

As mentioned previously, multiple preclinical and clinical reports that demonstrate not only the significance of CSCs in different types of cancer, metastasis, and recurrence but also suggest that targeted therapies against these cells may be effective (Desai et al., 2019). As shown Figure 2, a small number of CSCs within the total tumor burden is capable of reproducing the whole tumor mass, so that targeting these cells in addition to conventional therapy could help prevent tumor recurrence.

CSCs are extremely important in OC, and their targeted elimination may be an efficient approach to reduce resistance to chemotherapy and cancer recurrence (Walters Haygood et al., 2014). Targeting signaling pathways in OC could be used to attack CSCs. Because these signaling pathways are responsible for the increased survival of CSCs, disrupting the pathways could help eliminate the CSCs. In fact, the NOTCH signaling pathway contributes importantly to the maintenance and survival of CSCs (Barnawi et al., 2016). Another targeted therapy for ovarian CSCs could rely on the elimination of cell surface markers like CD44, CD24, CD133, and CD117. It was reported that CD133^+^ OVCAR5-luc cells could be eliminated, which significantly reduced tumor progression (Skubitz et al., 2013). Moreover, there are other treatment strategies available for the targeted elimination of ovarian CSCs relying on signaling pathways, surface markers, interfering with niches, miRNAs, or differentiation therapy (Keyvani et al., 2019).

Several CSC-specific markers and signaling pathways could be treatment targets for CSCs, although they have not been extensively studied in CC. Moreover, several therapeutic methods have been proposed and evaluated to attack CSCs (Sudhalkar et al., 2019). One new therapeutic approach involves CSC-targeted nanoparticles (NPs), in which the NPs are designed to affect stem cell-associated functions via targeting specific signaling pathways such as Notch, reactive oxygen species (ROS) signaling, CSC-specific markers, or Wnt/β-catenin. Also, these NPs may interfere with the maintenance of cell stemness (Wang et al., 2012; Yang et al., 2014; Dou et al., 2015; Hong et al., 2015; Kim et al., 2015; Rao et al., 2015; Rotherham and El Haj, 2015). Similarly, the targeting of endometrial CSCs could be used to improve the chance of a complete cure and prevent its recurrence. There are various strategies including targeting the surface markers of the cancer stem cells, including CD133+ and CD117+. Different molecular techniques can be used for targeting of these cells including molecular-targeted agents such as miR-199b-5p, the γ-secretase inhibitor DAPT, and inhibitors of the Notch pathway (Garzia et al., 2009; Hovinga et al., 2010). Some studies have reported the proapoptotic and antiproliferative effects of HDAC inhibitors on EC cells (Takai et al., 2004; Jiang et al., 2007; Ahn et al., 2008). Moreover, salinomycin has been shown to inhibit fibronectin expression and reduce the proliferation, migration, and invasion of endometrial CSCs (RK12V-SP and Hec1) (Lu et al., 2011).

## Cancer Stem Cell Markers and Molecular Targeted Therapies

CSC surface markers provide molecular targets for several cancers, allowing the use of therapeutic antibodies specific for CSC surface markers. Various CSC surface markers have been identified and published. Interestingly, most of the markers used to identify CSCs are derived from surface markers present on hESCs or adult stem cells (Kim and Ryu, 2017).

The development of therapeutic strategies to target CSCs mainly relies on the use of cell surface markers to identify, enrich, and/or isolate CSCs. Many CSC surface markers have been identified, although some surface markers are considered to be controversial and require further investigation. Interestingly, most of the current CSC surface markers are derived from known surface markers of normal embryonic or adult stem cells (Islam et al., 2015; Kim and Ryu, 2017). The similarity between cell surface markers suggests that CSCs predominantly originate from normal stem cells via the accumulation of epigenetic and genetic alterations. There are currently 40 published CSC surface markers, which are classified into three different categories, relating to hESCs, adult stem cells, and normal tissue cells (Kim and Ryu, 2017). The first group of CSC surface markers is expressed on hESCs but is weakly or rarely expressed on normal tissue cells. The second group of CSC surface markers is expressed on adult stem cells but is weakly or rarely expressed on normal tissue cells. The third group of CSC surface markers is expressed on hESCs and/or adult stem cells, and is also considerably expressed on several normal tissue cells.

CD133 is the most frequently studied CSC surface marker in many cancers, and specific antibodies/immunotoxins against CD133 have been successfully developed for the selective eradication of CSCs (Bach et al., 2013; Schmohl and Vallera, 2016). CD133 is also one of the most frequently studied surface markers in solid cancers. It appears that the level of CD133 protein expression does not alter upon cell differentiation; however, tertiary conformational changes in differentiated colon cancer cells block the binding of an anti-CD133 antibody, suggesting that the expression of the CD133 epitope is restricted to undifferentiated stem cells (Grosse-Gehling et al., 2013). CD117 is involved in signal transduction for survival and self-renewal in various cells (McLaughlin et al., 2007). Human epithelial OC CD44+CD117+ cells were shown to possess CSC properties, along with increased chemoresistance (Chen et al., 2013). LGR5 is a CSC marker in mouse intestinal cancer (Ahmed et al., 2010) and has also been suggested to be a CSC marker for human colon tissue and colorectal cancer (Kemper et al., 2012; Hirsch et al., 2014).

## Conclusion

Many efforts have been made to design therapeutic approaches that specifically target CSC populations. This is because CSCs have been recently predicted to be a crucial population to eliminate. However, recent insights have complicated the initially elegant model, by showing the dominant role for the tumor microenvironment in determining CSC properties. This is particularly important since the dedifferentiation of non-tumorigenic cancer cells to produce CSCs has been shown to occur, and therefore, the CSC population in a neoplasm can vary over time. Moreover, evidence suggests that not all tumors are driven by rare CSCs, but might instead contain a larger population of tumorigenic cells. Even though these results suggest that specific targeting of the CSC population might not necessarily be a useful therapeutic strategy, research into the hierarchical cellular organization of tumors has provided many important new insights into the biology of tumors.

Due to the deficiencies of conventional routine therapy of gynecologic cancer, especially recurrent and advanced stages, the development of specialized and more targeted therapeutic approaches could be very promising. Furthermore, accumulating data concerning genomic and proteomic profiling over the last several years have paved the way for understanding the molecular foundation of human cancer and the role of several genes, which have been altered, activated, or inactivated in tumors.

Therefore, the present review has summarized some advances in the targeted treatment for each molecular tumor profile. Importantly, CSCs are one of the current limitations in treatment of different cancers because of their resistance to chemotherapy drugs caused by different mechanisms. Therefore, more studies are needed to target these cells and to understand their functional mechanisms to prevent the recurrence of cancer.
